# Health system performance assessment landscape at the EU level: a structured synthesis of actors and actions

**DOI:** 10.1186/s13690-016-0173-5

**Published:** 2017-01-30

**Authors:** Nataša Perić, Maria M. Hofmarcher-Holzhacker, Judit Simon

**Affiliations:** 1 Department of Health Economics, Centre for Public Health, Medical University of Vienna, Kinderspitalgasse 15/1, 1090 Wien, Austria; 2 HealthSystemIntelligence, Josefstädterstraße 14, 1080 Wien, Austria

**Keywords:** Health system performance assessment, Health information, Policy making, International comparisons, European Union, Public health indicators

## Abstract

**Background:**

Many policy makers and other stakeholders in the EU have expressed interest in better understanding the performance of their own health systems to identify opportunities for improvement in effectiveness, efficiency and equity. Health system performance assessment (HSPA) has received considerable attention at EU level as an instrument to improve transparency and accountability. This is equally important for population health and sustainable health spending. The goal of this paper is to synthesise and map the current state and developments in the field of HSPA relevant in the EU context and by this aid the navigation in the growing HSPA system, understand the available tools and identify opportunities for improvement.

**Methods:**

Structured synthesis of the literature on initiatives in the field of HSPA at EU level was carried out. Key literature was identified by a focused review performed between October 2015 and June 2016 on websites of key institutions including the EU, OECD and WHO and Google engine. We used six predefined criteria for identifying key literature. Identified initiatives were classified according to analytical and conceptual output or whether a guiding or advisory role was resumed. A visual map of the relationships between the different actions and actors involved in HSPA was developed. In addition, expert opinion was sought to refine the map.

**Results:**

We identified a total of 64 relevant initiatives and their relationships in the field of HSPA. These include institutions such as the European Commission (73%), European Council (8%), OECD (9%) and WHO-EUR (9%). 24 initiatives produced analytical outputs, four developed conceptual outputs and six had a guiding role. The role of the EU in HSPA and collaboration with other key actors have intensified considerably since the adoption of the EU Health Strategy in 2013. The EU HSPA landscape is complex with seemingly few streamlining activities.

**Conclusions:**

Knowledge transfer and exchange of expertise are key to HSPA. While cooperation between the key actors have intensified recently and clearly reflect the “Health in all Policies” (HIAP) approach, there is considerable room for improved streamlining activities to share knowledge and avoid overlapping efforts, especially within the European Commission.

## Background

Many policy makers, researchers, public health specialists and other stakeholders in the European Union (EU) have expressed interest in better understanding the performance of their health systems to identify opportunities for improvement in effectiveness, efficiency and equity. At the same time, health system performance assessment (HSPA) at EU level has received considerable attention as an instrument to improve transparency and accountability.

According to Smith [1, 2] the prime objectives of HSPA are:

- to set out the goals and priorities for a health system;
- to act as a focus for policymaking and coordinating actions within the health system;
- to measure progress towards achievement of goals;
- to act as a basis for comparison with other health systems;
- to promote transparency and accountability to citizens and other legitimate stakeholders for the way that money has been spent.

While policy making in many areas of EU health systems is in the responsibility of Member States (MSs), cross-country comparisons of health system performance have become increasingly important. First, structural reform in many MSs and policy recommendations from the European Commission have been increasingly targeting health care. A large and growing share (30% in 2013) of social protection spending in the EU is used for health care services [3]. Second, the crisis including recent migrant waves led to increased pressure on various segments of social spending including health. Sustainability and efficiency of health spending is thus high on the agenda. The development of the directive aimed at making cross-border health care for the EU citizens possible is seen as another major driver behind the increased use of comparisons and benchmarking of quality of care provided [2, 4]. Also, the growing availability of comparable datasets has enhanced the technical feasibility to compare performance [2]. These data, carefully analysed and accompanied by conceptual models of knowledge are powerful tools to influence policy-making [2, 5]. Finally, providing expertise and assistance in the area of HSPA are a fundamental role for the EU level, in setting standards, collating and disseminating experience, and disseminating standardised information. Such endeavours are a public good that can only be done effectively and comprehensively at EU level, and can contribute new resources for guiding policy and practice in individual MSs.

Health system performance is measured against multiple objectives. This calls for a strong framework covering access, equity, efficiency and quality and their interrelation in order to understand the content and the scope of the comparison [2, 6]. Although HSPA is primarily a country-specific process for which there is no single accepted template, having harmonised methodologies and tools to support health policy makers in taking decisions requires actions also at EU level [1, 7]. The importance of good practice HSPA therefore has been receiving high-level support both at national and at EU level [7, 8]. The European Commission (EC) communication on effective, accessible and resilient health systems [9] and the mandate from November 2014 given to the European Commissioner for Health by the EC President to develop expertise for HSPA provide evidence of this support [10].

Progress has been made in monitoring the health of the population and the performance of health systems in terms of the scope, nature and timeliness of performance data that have been made publicly available over the last 30 years [11]. Nevertheless, methodological challenges remain in accomplishing the aim of health system governance improvement. These challenges include creating and unifying reporting standards of data and indicators, and establishing coherent HSPA frameworks for cross-country comparisons [12]. This should ultimately guide the way how best to deploy performance data [11]. The wider performance framework developed under the EuroREACH project based on the OECD HCQI initiative may be seen as one of the starting points in this context [6].

Clear evidence is needed on the prioritization, including the necessity and rationale for specific health system indicators. Furthermore, core indicators for different types of policy-use, (e.g. monitoring/forecasting, benchmarking, target-setting, cross-country comparison) should be identified and categorised accordingly. In recent years, some advanced cross-country approaches were established analysing the comparative performance of disease specific variables across countries (EuroHOPE) [13] as well as regional variations of hospital indicators (ECHO) [14]. However, there is a lack of a European-wide coherent framework addressing data needs, quality of data and guidance in using and applying indicators of HSPA.

While the Euro Health Consumer Index (EHCI), a private initiative ranking 35 European health systems according to their performance along 38 indicators shed light on the potential of comparing important health and health system indicators [15], it has been criticised by some for the lack of transparency in indicator selection and scoring [16]. More work is needed in providing standards for the quality of indicators and for a sound rational of monitoring specific indicators and their significance in cross-country comparisons. These challenges are being addressed in BRIDGE Health, a European project that aims at preparing a comprehensive, integrated and sustainable EU health information system which will incorporate know-how and technical tools to coordinate and harmonise research and surveillance for MSs in key EU health policy areas [17]. The present work forms an integral part of BRIDGE Health research activities. In addition, the BRIDGE Health System Indicator Task Force was established to exchange expertise on the work of health system performance indicators development.

Currently, stakeholders have difficulty navigating the HSPA landscape, understanding the opportunities and tools it provides, and using the data to identify opportunities for improvement in effectiveness, efficiency and equity of their healthcare systems. To the best of our knowledge, no synthesis exists to help understand the organization of the HSPA system at the EU level. Hence, the goal of this paper is to synthesise the current state and developments in the field of HSPA relevant to the EU context by visually mapping the relationships between the different actions and actors involved in HSPA in the EU.

The rest of the paper is organised as follows. The second part presents the methods, including six predefined selection criteria of initiatives and the output classification that led to the structure of the visual map. Section 3 describes the different initiatives that were identified both at EU and joint action levels. Finally, the implications of the current structure of HSPA in the EU are discussed.

## Methods

### Sources and initiative selection

A focused search was conducted and documented to identify initiatives in health system performance at the EU level. For simplicity, in this initial step we considered initiatives that are either completely or mostly at EU level covering the following key institutional players: the European Commission and its relevant Directorates-General (DG), EU agencies and the European Council, the Organisation for Economic Cooperation and Development (OECD), and the World Health Organisation Regional Office Europe (WHO-EUR). Initiatives developed at the level of MSs and other international or intergovernmental initiatives that do not pertain to Europe (e.g. Commonwealth Fund, World Bank) were excluded. Due to the nature of institutional websites as the primary source of this search and the inherent linkage between findings, formal systematic review methods were not pursued for the mapping exercise. The following selection criteria for initiatives were applied of which a minimum of three had to be fulfilled:

1. Initiatives that aim to harmonise monitoring of health systems and health policy: initiative i) use internationally comparative data, ii) are available within the last 16 years, iii) have recurrent output available in English language.
2. Initiatives that pertain to HSPA frameworks.
3. Initiatives that seek to foster transparency, accountability and accessibility of data in the field of public health and health systems research.
4. Initiatives that inform a blueprint for an indicator repository of a European health information infrastructure by referring to a list of indicators.
5. Initiatives that provide assessment of indicators and meta-information related to the provision of i) references on the indicator selection process, and ii) description, calculation, rationale, data availability, comparability of indicators.
6. Initiatives that provide analytical outputs for evaluating health systems including a quantitative analysis accompanied by an interpretation of analytical outputs.

The current search was conducted on over 25 websites of the key institutions, and the top 50 search hits of the Google website. In addition, the database on completed European and international projects drawn from the Health Data Navigator (HDN) was used [18]. The HDN was developed as part of the EuroREACH project and is continued in BRIDGE Health. The search was limited to a publication date after 2000 and was performed between October 2015 and June 2016 accompanied by three rounds of adjustments based on input from experts of the BRIDGE Health System Indicators Task Force. Included were policy and research documents, consultation and opinion papers, project reports, meeting minutes and other corresponding electronic documents. In total, over 75 websites were searched with the following terms in English to draw out a broad range of relevant initiatives: “health system performance assessment” AND (“framework” OR “initiative” OR “method”) AND (“EU” OR “European” OR “Europe” OR “European Commission”). Tthe search string (“EU” OR “European” OR “Europe” OR “European Commission”) was not used on websites related to the EC and the European Council to avoid redundancy. Reference lists of included literature were also examined. However, the majority of the initiatives included in this synthesis were identified with the search terms on the respective websites.

### Synthesis

A visual organizational map was created which features identified initiatives. Such a map is a commonly used diagram in the field of business administration that outlines the roles, responsibilities and relationships between individuals within an organization in just one picture [19, 20]. It typically illustrates the relations and decision making power by linking the different functions depicted as boxes with lines on different levels [19, 20]. For our purpose, the map was organised by institution and the extent of their contributions by making use of graphical forms and colour schemes. The map makes use of *angular rectangles* to represent these institutions, their associated key directorates the supporting agencies as well as the individual initiatives. We further distinguished the type of resources produced by these initiatives:

- Analytical output *(yellow)*: provision of health data series, indicators and/or any kind of analysis or accompanied by respective country reports.
- Conceptual output *(green)*: providing literature review and/or conceptual innovation (e.g. suggesting indicators, defining frameworks).
- Guiding or advisory, body *(blue)*: mandated to create recommendations and issue opinions on specific topics related to health information and HSPA.

Initiatives for which more than one type of output applies are represented by multi-coloured rectangles reflecting their multidimensionality. Lines indicate collaborative or supportive relations between actors and initiatives. While there are of course links between Eurostat and OECD, the map expresses this collaboration via the central role of the EC. A grey line connecting EC and OECD is used to represent this increasingly important collaboration. This synthesis is complemented by a timeline perspective in the form of a flow chart were the evolvement of actors and actions between 1998 and 2016 in the field of HSPA is displayed.

## Results

**Fig. 1:**
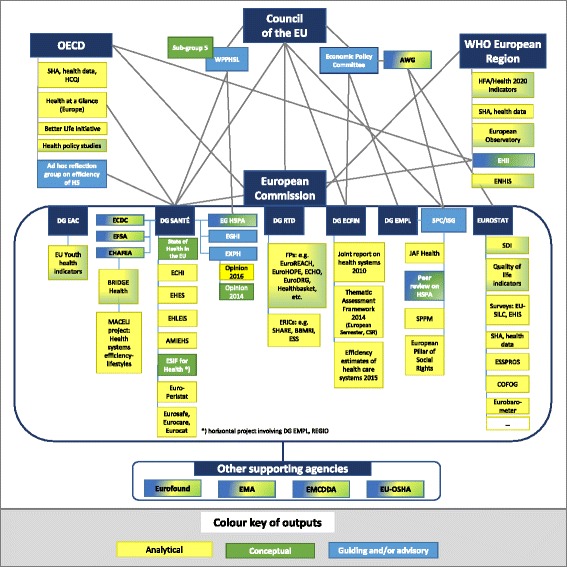
Existing HSPA initiatives at EU and at international level. Legend: **AWG**: Working Group on Ageing Populations and Sustainability; **BBMRI**: Biobanking and Biomolecular Resources Research Infrastructure; **CHAFEA**: Consumer, Health and Food Executive Agency; **COFOG**: Classification of the Functions of Government; **CSR**: Country Specific Recommendation; **DG EAC**: Directorate-General for Education and Culture; **DG ECFIN**: Directorate-General for Economic and Financial Affairs; **DG EMPL**: Directorate-General for Employment, Social Affairs and Inclusion; **DG RTD**: Directorate-General for Research & Innovation; **DG SANTÉ**: Directorate-General for Health and Food Safety; **ECHI**: European Core Health Indicators; **ECHO**: European Collaboration for Health Optimization; **EFSA**: European Food Safety Authority; **EGHI**: Expert Group on Health Information; **EHII**: European Health Information Initiative; **EHIS**: European Health Interview Survey; **EHLEIS**: European Health and Life Expectancy Information System; **EMA**: European Medicines Agency; **EMCDDA**: European Monitoring Centre for Drugs and Drug Addiction; **ENHIS**: Environment and health 22 indicators system; **ERIC:** European Research Infrastructure Consortium; **ESIF**: European Structural and Investment Funds; **ESS:** European Social Survey; **ESSPROS**: European system of integrated social protection statistics; **EU-OSHA**: European Agency for Safety and Health at Work; **EuroDRG**: Diagnosis-Related Groups in Europe: towards Efficiency and Quality; **EuroHOPE**: European Health Care Outcomes, Performance and Efficiency; **EuroREACH**: A Handbook to Access Health Care Data for Cross country Comparisons of Efficiency and Quality; **SHARE**: Survey of Health, Ageing and Retirement in Europe; **EU-SILC**: European Union Statistics on Income and Living Conditions; **EXPH**: Expert Panel on Effective Ways of Investing in Health; **HCQI**: Health Care Quality indicators; **HFA**: Health for All; **HS**: Health system; **HSPA**: Health System Performance Assessment; **ISG**: Indicators’ sub-group; **JAF**: Joint Assessment Framework; **MS**: Member State; **OECD**: Organisation for Economic Co-operation and Development; **SDI**: Sustainable Development Indicators; **SHA**: System of Health Accounts; **SPC**: Social Protection Committee; **SSPM**: Social Protection Performance Monitor; **Sub-group 5**: Sub-group 5 on measuring and monitoring the effectiveness of health investments; **WHO**: World Health Organisation; **WPPHSL**: Working Party on Public Health at Senior Level

**Fig. 2:**
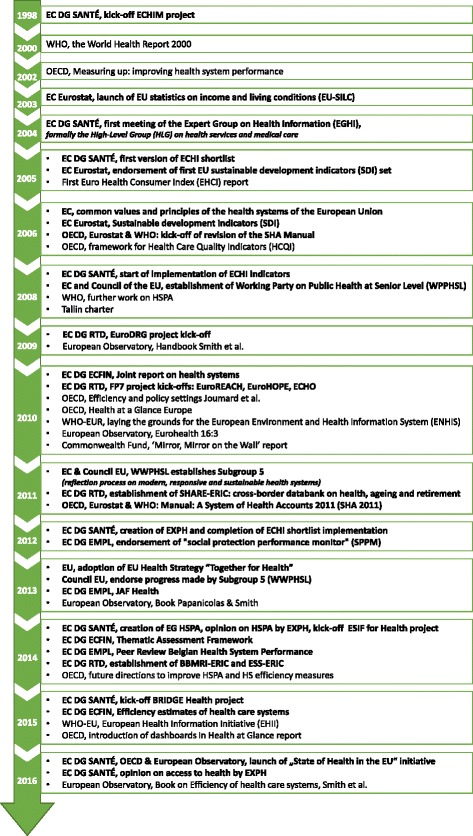
Evolvement of actors and actions in the field of HSPA, EU initiatives in bold. Legend: **BBMRI**: Biobanking and Biomolecular Resources Research Infrastructure; **COFOG**: Classification of the Functions of Government; **CSR**: Country Specific Recommendation; **DG EAC**: Directorate-General for Education and Culture; **DG ECFIN**: Directorate-General for Economic and Financial Affairs; **DG EMPL**: Directorate-General for Employment, Social Affairs and Inclusion; **DG RTD**: Directorate-General for Research & Innovation; **DG SANTÉ**: Directorate-General for Health and Food Safety; **ECHI**: European Core Health Indicators; **ECHO**: European Collaboration for Health Optimization; **EGHI**: Expert Group on Health Information; **EHIS**: European Health Interview Survey; **EHLEIS**: European Health and Life Expectancy Information System; **ENHIS**: Environment and health indicators system; **ERIC:** European Research Infrastructure Consortium; **ESIF**: European Structural and Investment Funds; **ESS:** European Social Survey; **EuroDRG**: Diagnosis-Related Groups in Europe: towards Efficiency and Quality; **EuroHOPE**: European Health Care Outcomes, Performance and Efficiency; **EuroREACH**: A Handbook to Access Health Care Data for Cross country Comparisons of Efficiency and Quality; **SHARE**: Survey of Health, Ageing and Retirement in Europe; **EU-SILC**: European Union Statistics on Income and Living Conditions; **EXPH**: Expert Panel on Effective Ways of Investing in Health; **HCQI**: Health Care Quality indicators; **HSPA**: Health System Performance Assessment; **JAF**: Joint Assessment Framework; **OECD**: Organisation for Economic Co-operation and Development; **SDI**: Sustainable Development Indicators; **SHA**: System of Health Accounts; **SSPM**: Social Protection Performance Monitor; **WHO**: World Health Organisation; **WPPHSL**: Working Party on Public Health at Senior Level

Figure 1 presents the current structure of HSPA initiatives and their respective organizational actors. The synthesis of these initiatives resulted in a total of 64 activities indicated by different boxes. It consists of a total of four key institutions aligned with six DGs in the EC which are linked to seven supporting agencies. The majority of displayed initiatives is associated with the EC and the European Council amounting to 37 in total when excluding the aforementioned EU institutional players (*N* = 17). Five initiatives were included for both the OECD and WHO-EUR. Concerning the type of output, the map displays a total of 24 initiatives that were classified as solely producing analytical outputs, four with conceptual outputs and six with a guiding and/or advisory role. Both analytical and conceptual outputs are provided by a total of nine initiatives which are represented by multi-coloured rectangles in green and yellow. A combination of conceptual output and guiding activities could be linked to a total of three initiatives illustrated by green and blue rectangles such as the “Peer review on HSPA” initiative. In total eight initiatives including the supporting agencieswere categorised to have all three types of output. Figure 2 gives an overview of the development process in the field of HSPA initiatives over time indicating their relevant starting dates. Brief narratives to the main findings are provided below.

### International initiatives

International agencies play an important role in promoting the comparison of health system performance across EU countries [2]. Since the World Health Report 2000, plenty of resources have been developed to facilitate such comparison and support HSPA in general [1]. The OECD and WHO have an instrumental role in driving international comparisons of health systems with their data repositories. The most important data are the OECD Health Statistics including the “Health Care Quality Indicators (HCQI)” [21–23] and the “WHO Health for All database” [24], and specific annual publications, such as “Health at a Glance” [25] or the “European Health Report” [26, 27]. Furthermore, we identified different HSPA related topics featured explicitly in publications resulting from the OECD’s “health policy studies” [28–31]. Analytical work on the efficiency of health care systems performed by Joumard and colleagues remains central in this area [29]. More recently, the OECD initiated an “ad hoc reflection group” to seek comments from experts on health care efficiency indicators with the intent that comments would facilitate prioritization of these indicators [32].

### Collaborative initiatives

A major milestone in the collaboration between the EC, OECD and WHO-EUR was the initiation of the joint data collection in the context of the OECD System of Health Accounts (SHA), the standard framework for producing consistent and internationally comparable health system financial data [1, 33]. Also, the biannual publication series “Health at a Glance: Europe” represents another major and vastly important collaboration output between the EC and OECD.The selection of indicators for this report is largely based on the “European Core Health Indicators (ECHI)” shortlist. This list has been created by the EC with the aim to monitor health in the EU [34]. The “European Observatory of Health Systems and Policies (OBS)” is a further partnership between the EC, the WHO-EUR, the World Bank and certain MSs serving as a prime source for informed and comparable descriptions of health systems [35]. The “European Health Information Initiative (EHII)”, established in March 2015, is another recent collaboration between WHO-EUR, EC, OECD, MSs and related health information networks and associations supporting the development of a single European health information system focusing on a set of targets and indicators as defined in Health 2020 [36]. The “State of Health in the EU” initiative, launched in June 2016, is the youngest collaborative initiative with DG Santé and OECD which is aided by the OBS. Itt aims to develop individual country health profiles to support health policies in EU countries [37].

### EU initiatives

Main actors within the European Commission in the field of HSPA are DG Santé, DG RTD, DG ECFIN, Eurostat, DG EAC and DG EMPL. The European Commission has created the “European Core Health Indicators (ECHI)” initiative, which assembles 88 indicators relevant to HSPA, for over 50 of which data are readily available and reasonably comparable. Besides the ECHI indicators and the joint “Health at a Glance: Europe” reports, DG Santé operates the field of HSPA with further initiatives. First, the “Expert Group on HSPA (EG HSPA)” serves as a forum where MSs exchange experiences on the use of HSPA at national level, and receive support in identifying tools and methodologies to develop HSPA further [38]. So far, one relevant report has been produced describing the different strategies MSs apply to assess quality of care. Further work of the EG HSPA will cover prioritised topics such as integrated and primary care [39, 40]. Furthermore, the independent and multidisciplinary “Expert Panel on Effective Ways of Investing in Health (EXPH)” also issued an opinion on identifying criteria for prioritizing areas for HSPA which was based on work of the EG HSPA’s predecessor, called “Sub-group 5 on measuring and monitoring the effectiveness of health investments” [12, 41]. The work of Sub-group 5 was initiated by the Council’s “Working Party on Public Health at Senior Level” in 2011. Most recently, access to health services was also subject to a detailed statement by the EXPH [42]. The “Expert Group on Health Information (EGHI)”, a consultative MSs body, supports the implementation of national and cross-EU health strategies including health information [43]. Core to this is the envisioned set-up of a European Research Infrastructure Consortium (ERIC) on Health Information which is currently being conceptualised within the BRIDGE Health project [43–45]. Fig. 1 lists most of the projects endowed by BRIDGE Health that collected new data and developed health information frameworks, standardisations and quality control methods. The “European Structural and Investment Funds ESIF for Health” developed a toolkit with a set of widely available indicators deemed useful for the final evaluation of actions supported from the ESIF [46].

Several other relevant projects have been funded under the Commission’s FP7 programme to identify and analyse health data from the perspective of cross-country comparisons including “EuroREACH” [47], “EuroHOPE” [13] and “ECHO” [48]. DG RTD funds EU research programmes as part of Horizon2020 and the Joint Research Centre (JRC). In the recent programme JRC aims to provide science and knowledge service on health and safe environment and consumer health and safety among other non-nuclear topics [49]. In addition, the “European Research Infrastructure Consortia (ERICs)” “SHARE”, “BBMRI” and “ESS” were established to provide resources and related services used by the scientific community to conduct top-level research in the broader field of health [50–53].

DG ECFIN has produced three relevant pieces of work focusing on the sustainability of health services. For example, in the context of the “European Semester” Country-Specific Recommendations (CSR) are made through the analysis of health system efficiency [54–57].

At EU level, the European Statistical Office, “Eurostat”, is the main data collection body and main data provider [58]. Its role in the area of high quality health data has enhanced greatly in recent years. In light of the broadened understanding about the importance of wellbeing for economic progress, the “Quality of life” indicators were developed [59]. Together with OECD’s “Better Life initiative” [60] they form a further strand of approaches where relevant indicators are being used. These initiatives are currently only loosely connected. At Eurostat level, the initiative on “Sustainable Development Indicators” is using selected health indicators at different levels of importance for monitoring the sustainable development strategy of the EU [61]. DG EAC monitors the health of Europe’s youth with one tailored tool [62, 63].

Lastly, the “Social Protection Committee (SPC)” and its Indicators Subgroup adapted the “Joint Assessment Framework (JAF)” methodology to the area of health systems. This serves as a reference HSPA tool for supporting the monitoring and assessment of structural reforms focusing on issues related to access, quality and equity [5, 8]. Progress on the social protection policy goals is monitored with a tool called “Social Protection Performance Monitor (SPPM)” that makes use of some specific ECHI indicators [64]. The recent “European Pillar of Social Rights” initiative led by DG EMPL also addresses the importance of timely access to ensure good quality and affordable health care and long-term care for Europeans [3].

## Discussion

This paper focused on synthesising and visualizing major HSPA activities and actions in the EU context initiated before and ongoing in June 2016. This synthesis is complemented by a timeline perspective. Fig. 2 gives an overview of the development process in the field of HSPA initiatives. It shows that activities in this area have intensified in recent years including enhanced efforts to better collaborate across key international actors. The results of this synthesis paper highlight three main points within the sphere of HSPA in the EU.

First, there is a clear reflection of the “Health in all Policies” (HIAP) approach [65] emphasised in the Commission’s Communication in 2014 on health systems and a growing visibility of HSPA as shown in the timeline perspective [9]. The Commission promotes cooperation at EU level with a view to strengthen effectiveness, increase accessibility and improve resilience of the national health systems in the EU. Many DGs have activities in place to promote HSPA, such as the provision of comparable indicators to monitor health and health systems (ECHI), expert bodies for better exchange of experiences between MSs (e.g. EG HSPA) or structured HSPA frameworks to guide monitoring (JAF Health). These actions are complemented by co-funded research in this area [44].

Second, the map reflects the complexity of coordinating recent activities in HSPA. Different roles assumed by individual DGs and their relationships between each other within the field of HSPA are not entirely clear at this stage. For example, while DG Santé is mainly concerned with population health and health information issues in general, DG EMPL has a focus on access to health care including equity issues, and DG ECFIN concentrates on efficiency and sustainability. While this fragmentation between DGs largely reflects policy mandates under current EU governance responsibilities, it calls for improved cooperation across them to better bundle expertise on the different domains covered by HSPA. Also and as Fig. 2 indicates, HSPA only gained formal attention in 2013 with the adoption of the EU Health Strategy “Together for Health” [66]. Even though the Commission managed to considerably increase the visibility of the role of the EU in HSPA since then, the visualisation of the current activities suggests that coordination needs improvement. For example, to achieve the goals outlined in key documents on health systems 2014 regarding e.g. resilience and sustainability likely requires more capacities across Commission services [9, 10]. Increasing cooperation within the Commission would reduce duplications of work and ensure more inclusivity.

Third, the current mapping exercise should be seen as a starting point for extension to MS-level HSPA initiatives and potential other relevant international initiatives to build the base for the development of a comprehensive indicator repository. Such initiatives will be included in the planned next wave of our HSPA research in the context of BRIDGE Health activities. The work will focus on a comprehensive synthesis of the relevant indicator landscape. Starting point is the development of the european Health System Indicators survey (euHS_I survey). The euHS_I survey aims to identify overlaps between these initiatives and gaps in the relevant availability of health care system performance indicators. Also, the importance of indicators for the different performance domains will be assessed. This should lead to the compilation of a set of high quality headline indicators with important policy relevance for HSPA.

The synthesis provided within this paper has limitations. First, it focuses on major players and excludes MS initiatives as well as other potentially relevant international initiatives (e.g. World Bank, EHCI, Commonwealth Fund). Second, the visual map shows the status quo in 2016 including also bodies that were replaced by newly created Expert groups such as the Sub-group 5 from the WPPHSL paving the way to the creation of the EG HSPA. Figure 2 was created to complement the map with a timeline. However, communication/collaboration lines between different DGs were omitted for simplification purposes. For example, there is cooperation and exchange between EG on HSPA with EGHI and SPC/ISG. Not displaying those lines should not create the impression that there is no collaboration or interaction at all.

## Conclusions

This paper synthesises, maps and describes the current developments in HSPA at EU level. To our knowledge, this is the first piece of work to map out the relationships between different actors and actions driving the field of HSPA.

The results of this mapping exercise can be used to obtain a comprehensive overview of the current developments in the field of HSPA. In summary, our findings indicate that the European Commission’s role in HSPA is increasingly important to promote standards for good practice HSPA. Also, activities are largely consistent with goals outlined with the adoption of the EU Health Strategy in 2013. This reflects the strengthened role of the European Commission as a hub for promoting unified standards to enhance comparative analysis of welfare systems. Equally, it acknowledges the increasing importance of health system performance for European policy making. However, the coordination of various HSPA activities by different actors, especially within the European Commission leaves room for improved cooperation in order to avoid duplication of work and ensure overall efficiency of ongoing activities.

## Abbreviations

AWG: Working Group on Ageing Populations and Sustainability
CHAFEA: Consumer, Health and Food Executive Agency
COFOG: Classification of the Functions of Government
DG EAC: Directorate-General for Education and Culture
DG ECFIN: Directorate-General for Economic and Financial Affairs
DG EMPL: Directorate-General for Employment, Social Affairs and Inclusion
DG RTD: Directorate-General for Research & Innovation
DG SANTÉ: Directorate-General for Health and Food Safety
ECDC: European Centre for Disease Prevention and Control
ECHI: European Core Health Indicators
ECHO: European Collaboration for Health Optimization
EFSA: European Food Safety Authority
EGHI: Expert Group on Health Information
EHII: European Health Information Initiative
EHIS: European Health Interview Survey
EHLEIS: European Health and Life Expectancy Information System
EMA: European Medicines Agency
EMCDDA: European Monitoring Centre for Drugs and Drug Addiction
ENHIS: Environment and health indicators system
EPC: Economic Policy Committee
ESIF/ESI Funds: European Structural and Investment Funds
ESS: European Social Survey
ESSPROS: European system of integrated social protection statistics
EU: European Union
EU-OSHA: European Agency for Safety and Health at Work
EuroDRG: Diagnosis-Related Groups in Europe, towards Efficiency and Quality
EuroHOPE: European Health Care Outcomes, Performance and Efficiency
EuroREACH: A Handbook to Access Health Care Data for Cross country Comparisons of Efficiency and Quality
EU-SILC: European Union Statistics on Income and Living Conditions
EXPH: Expert Panel on Effective Ways of Investing in Health
HCQI: Health Care Quality indicators
HFA: Health for All
HIAP: Health in all Policies
HIREP-ERIC: European Research Infrastructure Consortium on Health Information for Research and Evidence-based Policy
HS: Health system
HSPA: Health System Performance Assessment
ISG: Indicators’ sub-group
JAF: Joint Assessment Framework
MS: Member State
OECD: Organisation for Economic Co-operation and Development
QoL: Quality of Life
SDI: Sustainable Development Indicators
SHA: System of Health Accounts
SHARE- ERIC: Survey of Health, Ageing and Retirement in Europe - European Research Infrastructure Consortium
SPC: Social Protection Committee
SSPM: Social Protection Performance Monitor
WHO: World Health Organisation
WPPHSL: Working Party on Public Health at Senior Level
